# The Role of Social Skills in Predicting Treatment-Recovery in Children with a Social Anxiety Disorder

**DOI:** 10.1007/s10802-021-00824-x

**Published:** 2021-06-24

**Authors:** Anke M. Klein, Juliette M. Liber, Natasja D. J. van Lang, Catrien Reichart, Maaike Nauta, Brigit M. van Widenfelt, Elisabeth M. W. J. Utens

**Affiliations:** 1 Developmental and Educational Psychology, Leiden University, Leiden, The Netherlands; 2 Clinical Child and Adolescent Psychology, Ruhr University, Bochum, Germany; 3 Developmental Psychology, Utrecht University, Utrecht, The Netherlands; 4 Curium, Leiden University Medical Center, Leiden, The Netherlands; 5 Faculty of Behavioural and Social Sciences, University of Groningen, Groningen, The Netherlands; 6 Research Institute of Child Development and Education, University of Amsterdam, Amsterdam, The Netherlands; 7 Academic Center for Child Psychiatry the Bascule/AMC, Amsterdam, The Netherlands; 8 Department of Child and Adolescent Psychiatry, Erasmus University, Rotterdam, The Netherlands

**Keywords:** Childhood anxiety, Social anxiety disorder, Social skills, Cognitive behavioral treatment (CBT)

## Abstract

The current study investigated the role of social skills and its interaction with social anxiety as predictors of treatment outcome in children with an anxiety disorder either with or without a social anxiety disorder (SoAD). In total, 133 children (aged 8 to 13) with an anxiety disorder received a 10-session cognitive behavioral treatment (FRIENDS program). Pre- to post treatment Reliable Change (RC) and Treatment-Recovery (TR) were assessed from a multi-informant perspective, by including diagnostic information (ADIS C/P), child-reported anxiety symptoms (MASC) and parent-reported internalizing symptoms (CBCL-Int). Social skills were assessed with the parent-rated Social Skills Rating System (assertion, self-control, responsibility). Results showed that 1) parents of children with a SoAD reported significantly less favorable use of assertive and responsible social behavior in their children pre-treatment than parents of children without SoAD, 2) children with higher social skills had a better treatment recovery, and 3) children with anxiety and higher responsible behavior pre-treatment and without a SoAD had a better treatment recovery, but this effect did not show for children with SoAD. In conclusion, better use of social behavior increased the likelihood of treatment recovery but not of reliable change. Further studies on the role of social skills in the treatment of childhood (social) anxiety are needed to investigate the mechanisms by which social skills impact treatment outcome.

Over the years, evidence has accumulated that cognitive behavioral therapy is effective for the treatment of childhood anxiety disorders. Most treatment outcome studies (for childhood anxiety) are eclectically approached and include a combination of evidence-based techniques (Emmelkamp et al., 2010; Fréchette-Simard et al., 2018). Offering children with various anxiety disorders an eclectic or generic treatment program may result in positive outcomes for the group in general (e.g., Nauta et al., 2003; Khanna & Kendall, 2010; for an overview, see Rapee et al., 2009; for a meta-analysis, see James et al., 2013), but may systematically result in less favorable outcomes for subgroups. For example, some recent studies suggest that children and adolescents with social anxiety have the poorest outcomes following treatment when compared to other anxiety disorders (e.g., Baartmans et al., 2019; Hudson et al., 2015a, b; Kodal et al., 2018; Lundkvist-Houndoumadi & Thastum, 2017; Waters et al., 2018). Indeed, more and more studies suggest that offering tailored / personalized treatment may result in better treatment outcomes (McLeod et al., 2013; Hudson et al., 2015b). In order to take the next step in improving treatment outcome, there is a serious need to identify factors associated with treatment success and failure especially in children with a social anxiety disorder (SoAD).

One of the candidate predictors for outcome of SoAD treatment that is often mentioned is *social skills* (e.g., Spence & Rapee, 2016). Social skills can be conceptualized as a specific class of behaviors that an individual exhibits in order to successfully complete a social task (e.g., communication, play, work together; McDaniel et al., 2017). Children showing difficulties with adequate social or interpersonal behavior are more likely to develop emotional or behavioral disorders (EBD), peer relationship problems, teacher-student relationship problems (Hebert-Myers et al., 2006). The adequate use of social skills plays an important role in day-to-day communication and interaction. As a result, children who show less adequate behavior in social situations may profit less from treatment as many of in-session activities and homework assignments involve communication and interaction with others (e.g., therapist, parents, peers, etc.), Moreover, these children might benefit less from treatment; their poor skills may hinder experimentation with and generalization of newly learned skills.

Poor social skills, including the use of inadequate social behavior is clearly linked to the maintenance and possibility also to the etiology of childhood social anxiety (for a review and theoretical model of childhood social anxiety, see Spence & Rapee, 2016). Results on the relation between social anxiety and social skills are contradictory. Some studies conclude that children with social anxiety have a social skills deficit (Alfano et al., 2006; Dodd et al., 2011; Ginsburg et al., 1998; Inderbitzen-Nolan et al., 2007; Miers et al., 2010; Spence et al., 1999). Other studies do find that children with social anxiety behave less adequately in social situations than their non-anxious peers and conclude that this difference might not be due to a social skills deficit per se, but that children with social anxiety have difficulty to adequately use their social skills due to their anxiety (see also, Hopko et al., 2001; Spence & Rapee, 2016). Some studies even shed doubt on the relation between social anxiety and lower social skills and state that it only a self-perceived deficit and not an actual deficit (Cartwright-Hatton et al., 2003, 2005; Klein et al., 2018; van Niekerk et al., 2017). For example, Dodd et al. (2011) found that children with a social anxiety disorder had significantly lower ratings of social skills by observers than non-anxious controls. In other studies, evidence was found for impairments in social functioning and poorer social skills in children with social anxiety according to the parents (Ginsburg et al., 1998), and for a social skills deficit in adolescents with social anxiety compared to non-anxious adolescents (Inderbitzen-Nolan et al., 2007). Children with SoAD were also found to be less socially competent by their peers and were less likely to receive positive evaluations from peers during behavioral observations (Spence et al., 1999). On the other hand, Cartwright-Hatton et al. (2003) did not find evidence for poor social skills in children with social anxiety. The authors found that social anxiety was only weakly related to objective ratings of social skills, and that objective observers could not distinguish children with low levels of social anxiety from children with high levels of social anxiety when they participated in a discussion with an unfamiliar adult. Nevertheless, children with high levels of social anxiety rated their own social skills lower than peers with lower levels of anxiety.

In conclusion, there is evidence for the relation between social anxiety and displaying less adequate social behavior in different contexts, but it remain unclear if this is due to an actual social skill deficit, a performance deficit (i.e., they do have the social skills, but have problems to use it due to their social anxiety), or only a perceived deficit (i.e., they do report lower skills, but external raters do not see this). Despite the contradictory results, there is a clear consensus that children with social anxiety perceive themselves as being less socially competent which influences the way they perceive themselves, others and the world around them. Parent-reports on observed social behaviors may therefore better reflect children’s levels of social skills. To the best of our knowledge, there are no studies yet that compared parent reported social skills between children with social anxiety and children with anxiety but without social anxiety. The negative self-perception of children with social anxiety, and possibly also the difficulty to use their skills or even a deficit in their skills may have impact on the therapeutic process and could lead to greater variance in treatment outcome compared to children with anxiety but without social anxiety.

The overall aim of the current study was to investigate the role of parent-reported social skills performance on treatment outcome and the possible interaction with social anxiety. This study follows up on a study by Liber et al. (2008) who investigated the effect of individual versus group therapy in a large sample of children with an anxiety disorder and additionally also studied the effects of social anxiety on treatment outcome. They found comparable treatment outcome effects compared to other studies. In addition, no significant differences between group- and individual therapy and also no effects of social anxiety, nor interaction effects of social anxiety with group- versus individual therapy were found in this study. In this study, they measured social skills prior to treatment and mention that social skills may be an important factor in the treatment outcome especially for children with social anxiety, but they did not test this in their study. Therefore, the current study reanalyzed this data to examine 1) whether parents of children with SoAD reported different levels of social skills in their children prior to treatment than parents of children with an anxiety disorder without SoAD, 2) whether levels of social skills predicted treatment outcome, and 3) whether there was an interaction between parent-reported social skills and social anxiety. As there are, to the best of our knowledge, no studies that examined differences in parental reported social skills between children with SoAD and children with other anxiety diagnoses, we tested the hypothesis that SoAD is associated either with lower social skills or not within the sample of children with anxiety (with and without SoAD). We expected that especially children with lower social skills and a social anxiety disorder would have a significantly lower treatment outcome than children with higher social skills and an anxiety disorder without social anxiety in their profile (see also, Hofmann, 2000).

## Methods

### Participants

The sample was selected from consecutive referrals of 8–12-year-old children to the anxiety and depression unit of the outpatient university clinic for child and adolescent psychiatry of Leiden University Medical Center (LUMC)/ Curium or Erasmus Medical Center Rotterdam/Sophia. The present study was part of a study evaluating the efficacy of individual versus group cognitive behavioral treatment that ran between 2002–2006 (see also, Liber et al., 2008). As part of the routine procedure, children and their parents were interviewed with the Dutch version of the Anxiety Disorders Interview Schedule for children (ADIS-C/P; Siebelink & Treffers, 2001; Silverman & Albano, 1996). Children who received a diagnosis of social anxiety disorder (SoAD) separation anxiety disorder (SAD), generalized anxiety disorder (GAD), or specific phobia (SP) were included. A detailed description of exclusion criteria can be found in the study by Liber et al. (2008). A total of 142 children diagnosed with an anxiety disorder and their parents were asked to participate of which 133 participants signed informed consent and started treatment (59 girls; Age: *M* = 10.10, *SD* = 1.27). The treatment completers sample included 124 children since nine children (6.8%; 3 girls) dropped out of treatment. Of the resulting 124 children, 65 were treated individually and 59 children were treated in group format. In total, information was obtained from 123 mothers and from 108 fathers. Seven children did not maintain contact with their fathers or the fathers were unknown. Six fathers refused to participate, one father and one mother died, and two fathers lacked sufficient proficiency in Dutch. Demographic data are presented in Table 1. The committees for medical ethics of Leiden University Medical Center and of Sophia Childrens Hospital/ Erasmus Medical Center approved this study (ISRCTN48511871). The trial was registered at the Dutch Trial Register (NL309/ NTR 347).

**Table 1:** Demographic Data

	Children with SoAD (*n* = 39)	Children without SoAD (*n* = 85)
Age in years	*M* = 10.38 (*SD* = 1.24)	*M* = 9.92 (*SD* = 1.28)
SES Low Middle High	*n* = 7 *n* = 21 *n* = 11	*n* = 12 *n* = 36 *n* = 37
Primary diagnosis pre-treatment		
SoAD SAD GAD SP	*n* = *20* *n* = 12 *n* = 4 *n* = 3	*n* = *0* *n* = 38 *n* = 32 *n* = 15
Comorbidity N°D	*M* = 2.46 (*SD* = 1.19)	*M* = 1.62 (*SD* = 0.80)
Primary diagnosis post-treatment		
None SoAD SAD GAD SP	*n* = 16 *n* = 11 *n* = 7 *n* = 2 *n* = 3	*n* = 40 *n* = 3 *n* = 17 *n* = 9 *n* = 16

N^o^D   Mean number of disorders (e.g., Anxiety, ADHD, Depression), *SoAD* Social Anxiety Disorder, *SAD* Separation Anxiety Disorder, *GAD* Generalized Anxiety Disorder, *SP* Specific Phobia

### Measures

#### Multidimensional Anxiety Scale for Children

 (MASC; March, 1997; March et al., 1999). The MASC is a child self-report measure of pediatric anxiety for children aged 8 to 18 and includes 39 items which are rated on a 4-point-Likert-style scale. A Dutch translation of the MASC demonstrated an excellent internal consistency for the total score (α = 0.93) and a good re-test reliability (*r* = 0.81; Utens & Ferdinand, 2000). Internal consistency in the current study was excellent (T1: *α* = 0.90; T2: *α* = 0.92).

#### Child Behavior Checklist 

(CBCL; Achenbach, 1991; Verhulst et al., 1996). The CBCL is a standardized measure of parental perception of their children's emotional behavioral problems. For the current study, only the internalizing scale of the CBCL (CBCL-Int) was used. Good reliability and validity were found for the CBCL in a Dutch sample with cross-national correlations between 0.82 and 0.99 (de Groot et al., 1994). Internal consistency in the current study was good as well (mother: T1: *α* = 0.88, T2: *α* = 0.92; father: T1: *α* = 0.88, T2: *α* = 0.90).

*Social Skills Rating System* (SSRS; Gresham & Elliott, 1990). The SSRS is a parent-report measure that assesses social skills in their children by documenting the perceived frequency of social behaviors. The scale is specifically designed for children and consists of 38 items divided into 4 subscales: ‘assertion’ (i.e., asking others for information, introducing oneself, and responding to the actions of others), ‘self-control’ (i.e., behaviors that emerge in conflict-situations, such as responding appropriately to teasing, and in non-conflict situations that require taking turns and compromising), ‘responsibility’ (i.e., cleans up after him/herself, helps in the household on its own before asking help), and ‘cooperation’ (i.e., helping others, sharing and complying with rules and directions). The SSRS parent version proved to be reliable and valid (reliability coefficients range from 0.65 to 0.90 in a population of American children; Gresham & Elliott, 1990). A translation of the SSRS was developed at the department of child and adolescent psychiatry of the Sophia Children’s Hospital. To examine the psychometric properties of the SSRS data in a Dutch sample, we used data on a typical population collected as part of the TRAILS-study (Tracking Adolescents Individuals Lives Survey; De Winter et al., 2005). Exploratory factor analysis using the TRAILS data (*N* = 2230) suggested minor changes with regard to item distribution: The item-distribution for the scales ‘self-control’ and ‘responsibility’ was identical to the item-distribution of the original scale, while one item was added to the ‘assertions’ scale (item 27 ‘Gives compliments to friends or other children in the family. This item was originally included in the scale Cooperation’). The scale ‘cooperation’ showed the least similarities and was therefore excluded. For the current study, the subscales assertion, self-control, and responsibility were administered. Sufficient to good internal consistency showed for the three scales for both father- and mother reports (mother report: assertion: *α* = 0.77, self-control: *α* = 0.77, responsibility: *α* = 0.69; father-report: assertion: *α* = 0.79, self-control: *α* = 0.78, responsibility: *α* = 0.68). Mother- and father ratings in the current study correlated significantly (assertion: *r* = 0.74, *p* < 0.001; self-control: *r* = 0.63, *p* < 0.001; responsibility: *r* = 0.61, p < 0.001). As there were no statistically significant differences between the two raters (all *p-values* > 0.1) on all subscales, three combined mean subscale scores were computed. To adjust for gender effects, the subscales were standardized into t-scores using norm-data on boys and girls.

#### Anxiety Disorders Interview Schedule

 (ADIS-C/P; Silverman & Albano, 1996; Dutch translation: Siebelink & Treffers, 2001). The ADIS-C/P for DSM-IV was administered pre- and post-treatment to obtain clinical information from the parents and the child. The ADIS-C/P is a reliable instrument for deriving DSM-IV anxiety disorder symptoms and diagnoses in children aged 7 to 16. The interview is organized according to DSM-IV criteria and yield kappa coefficients for SoAD, SAD, SP and GAD in the good to excellent range for both the child- and the parent interview (Silverman et al., 2001). Pre-treatment ADIS-C/P interviews were conducted by licensed psychologists. During the conduct of the study clinicians and researchers met several times to ensure that procedures and decision making were alike. At post-treatment, interviews were conducted by trained master-students and researchers from the team (for a detailed description, see also Liber et al., 2008). Master level students were trained by observing live and videotaped interviews and completed an exam to prove acceptable administration of the interview. The interview reports were reviewed, supervised and discussed with the master students during the conduct of the study to ensure that administration, scoring and reporting would not drift.

### Treatment and Procedure

First, children and their parents were assessed with the ADIS-C/P. If all inclusion criteria were met and informed consent was obtained, children were assigned to either individual or group cognitive behavioral therapy by sequential randomization. All pre-treatment measures were administered in both parents and children. A waiting list condition was not used, since there is strong evidence that cognitive behavioral therapy is more effective than a waitlist condition (e.g., Cartwright-Hatton et al., 2004). One week post-treatment, children and parents were interviewed with the ADIS-C/P and assessment measures (including the MASC and the CBCL-Int) were again administered in both children and parents.

Children were treated with the FRIENDS program (Barrett & Turner, 2000; Dutch translation: Utens et al., 2001). This program is based on a theoretical framework with three main target areas for change: Physical symptoms, cognitive processes and coping skills. Several therapeutic techniques were used, such as cognitive restructuring, reinforcement, exposure and relaxation. All children received a manual-based 10-session weekly cognitive behavioral treatment program and two booster sessions, either in individual- or group format. Parents received 4 sessions of parent training, which included mainly psycho-education, also either in the group treatment or in the individual setting. If children were randomized to ICBT parent session were conducted individually, if children were randomized to GCBT parent session were conducted in groups. The treatment did not include social skills components per se, but rather stressed the importance of social support and included exercises that encouraged children to seek social support.

### Data Analysis

#### Treatment Outcome

An important issue related to the study of treatment outcome is a clear description of treatment success and treatment failure. The issue of statistically significant change and clinically meaningful change has been discussed repeatedly (Chambless & Hollon, 1998; Kazdin, 1999; Creswell, 2020). Various strategies to describe clinical significance have been suggested (Jacobson et al., 1999), and leaded to reporting clinically significant and meaningful change (e.g., Shortt et al., 2001; Silverman et al., 1999). When studying predictors for treatment outcome, researchers tend to fall back on commonly used strategies, such as regression analysis with raw scores (see also, Rapee, 2000; Victor et al., 2006). Using this kind of strategies lead to a model that accounts for variance in outcome, but not for clinically significant or meaningful change. Therefore, in the current study, outcome was defined in two ways: Pre- to post-treatment change (Reliable Change; RC, continuous variable) and success and failure (Treatment-Recovery; TR, dichotomy).

Post-treatment diagnostic status, as assessed with the ADIS C/P, reflected the clinical point of view on TR. Any references to treatment success based on ADIS-TR in the text from here on will indicate that children were free of *any* anxiety disorder at post-treatment according to consensus clinician composite rating based on child and parent interviews (see Creswell et al., 2020). Child self-reported anxiety symptoms (MASC) and parent-reported internalizing symptoms (CBCL-Int) at pre- and post-treatment reflected the point of view on TR and RC from the child, father and mother. TRs and RCs were computed with the procedure proposed by Hageman and Arrindell (1999) resulting in a modified Clinically Significant change Index (CSI; Jacobson & Truax, 1991). The RC-score is a continuous variable reflecting the amount of change. In order to calculate TR, several steps were taken. First, the RC-score was recoded into an index with three categories (deteriorated, not reliably changed, improved). Next, a score can be calculated indicating Clinical Significance, i.e. individual reliable passing of the cutoff for clinical significance (CS). The combination of the RC and CS-scores results in a fourth category: improved and recovered. The TR-score reflects a dichotomy of ‘recovered’ (fourth category) versus ‘not/partially recovered’ (combination of the categories deteriorated, no reliable change, improved but not recovered). In sum a client whose RC-score indicates improvement (RC < -1.65) *and* whose CS-score indicates individual reliable passing of the cut-off for clinical significance is considered to have recovered. The use of a dichotomy was applied to facilitate cross-battery comparison with the ADIS-C/P definition of treatment recovery.

#### Data-Analysis

The first aim of this study addressed the question whether children with SoAD versus children with an anxiety disorder other than SoAD differed with respect to the three subscales measuring social skills. T-tests were used to address this question. The second and third questions aimed to test the predictive value of social skills for treatment outcome (aim 2) and the potential interaction effects of social skills x SoAD for treatment outcome (aim 3). To address both research questions, we conducted a series of hierarchical regression analysis. We calculated separate analyses for each social skills subscale (assertion, self-control, responsibility), due to multicollinearity of the three subscales (assertion: Tolerance ranged between 0.35 and 0.55, VIF ranged between 1.98 and 2.83; self-control: Tolerance ranged between 0.34 and 0.65, VIF ranged between 1.54 and 2.94; responsibility: Tolerance ranged between 34. and 0.50, VIF ranged between 1.98 and 2.94). For the reliable change (RC) scores, we calculated separate linear regression analyses with MASC-RC and mother and father reported CBCL-RC-Int as dependent variables respectively, with pre-treatment subscales of social skills as a predictor in the first step, and we added SoAD and the interaction-effect between SoAD and social skills as additional predictors in the second step. For the treatment recovery (TR) scores, we calculated separate logistic regression analyses with the MASC-TR, mother and father reported CBCL-TR-Int and ADIS-TR as dependent variables respectively and pre-treatment subscales of social skills, and we added the interaction-effect between SoAD and social skills in the second step.

## Results

### Preliminary Analyses

Comparisons between children who completed treatment (*n* = 124) and non-completers (*n* = 9) showed that the two groups did not differ significantly on pre-treatment social economic status (χ^2^ = 1.98, df = 2, *p* = 0.371), age (*t* (131) = -1.06, *p* = 0.291), gender (χ^2^ = 0.41, df = 1, *p* = 0.520), primary diagnosis (χ^2^ = 0.41, df = 3, *p* = 0.938), parental reported social skills (mother: assertion; *t*(127) = -0.15, *p* = 0.884, self-control; *t*(127) = 0.99, *p* = 0.323, responsibility; *t*(127) = 1.19, *p* = 0.237; father: assertion; *t*(109) = 0.36, *p* = 0.722, self-control; *t*(109) = 0.33, *p* = 0.742, responsibility; *t*(109) = 0.88, *p* = 0.379), MASC (*t*(127) = 0.40, *p* = 0.693) or CBCL-Int (mother; *t*(122) = 0.32, *p* = 0.754, father; *t*(100) = 0.23, *p* = 0.822). Therefore, we assumed there were no drop-out biases and used the sample of treatment completers for the current paper.

Chi-square-tests were conducted to examine whether MASC-TR, mother and father reported CBCL-TR-Int and ADIS-TR scores were different in the individual versus the group treatment: The Chi-square values were all low and non-significant (ADIS-TR: χ^2^ (1) = 1.74, *p* = 0.188; MASC-TR: χ^2^ (1) = 0.01, *p* = 0.909; CBCL-TR-Int mother: χ^2^ (1) = 0.06, *p* = 0.813). Although results were significantly different for fathers (CBCL-TR-Int father: χ^2^ (1) = 4.41, *p* = 0.036), the continuity correlation was not significant (*p* = 0.095), one cell had an expected count below the threshold of 2,54. RC scores were not significantly different for children participating in individual treatment versus the group treatment (RC CBCL-Int mother; *t* (115) = 0.61, *p* = 0.543, RC CBCL Int father; *t* (90) = 0.53, *p* = 0.597, RC MASC; *t* (116) = 0.28, *p* = 0.778). We therefore analyzed all children as one group.

### Pre-Treatment Differences in Social Skills for Children with and without Social Anxiety

Comparison of pre-treatment parent-reported social skills in children with and without SoAD showed significant differences for the subscales assertion *t*(120) = 5.06, *p* < 0.001 and responsibility *t*(120) = 5.08, *p* < 0.001, but not for self-control *t*(121) = 0.91, *p* = 0.367 (see Table 2): Children with SoAD were described by their parents as less assertive and less responsible than children with an anxiety disorder without SoAD prior to treatment.

**Table 2:** Social Skills Rating System (SSRS), father and mother CBCL Internalizing and child MASC separately for clinically anxious children with and without a social anxiety disorder

	Children with SoAD (*n* = 39)		Children without SoAD (*n* = 83)			Children with SoAD vs children without SoAD
Measure	Mean	SD	Mean	SD		Cohens *d*
SSRS scales Parent						
Assertion	12.07	(2.83)	15.34	(3.53)		1.03
Self-control	10.01	(2.62)	10.57	(3.37)		0.19
Responsibility	10.58	(2.20)	13.14	(2.77)		1.03
CBCL Internalizing						
Mother Pre	22.11	(10.19)	19.10	(8.59)		
Mother Post	16.97	(9.70)	14.65	(9.16)		
Father Pre	15.81	(7.05)	16.21	(8.75)		
Father Post	14.01	(8.49)	13.47	(8.63)		
MASC						
Pre	43.20	(19.08)	39.81	(15.91)		
Post	37.73	(19.20)	36.42	(17.91)		

Means (SD), *SoAD *children with a social anxiety disorder pre-treatment

### Predicting Treatment Outcome

In order to predict treatment outcome by social skills (aim 2) and the interaction of social skills with SoAD (aim 3), we first calculated a series of hierarchical regression analyses.

#### Reliable Change (RC) scores

 For the RC scores, we found that parent-reported assertion, self-control, and responsibility and the interaction ‘Self-control x SoAP’ did not significantly predict the three RC variables (MASC: assertion *F*(1,114) = 0.23, *p* = 0.632, self-control *F*(1,115) = 2.92, *p* = 0.090, responsibility *F*(1,114) = 0.07, *p* = 0.789; RC CBCL Int father: assertion *F*(1,88) = 0.08, *p* = 0.780, self-control *F*(1,89) = 0.01, *p* = 0.908, responsibility *F*(1,88) = 0.00, *p* = 0.978; RC CBCL Int mother; assertion *F*(1,113) = 0.31, *p* = 0.579, self-control *F*(1,114) = 1.650, *p* = 0.202, responsibility *F*(1,113) = 0.43, *p* = 0.513). This means that parental reported level of social skills in children and its interaction with social anxiety was not related to change during treatment as reported by the children themselves, their parents and therapists.

#### Treatment- Recovery (TR) scores

 We calculated a series of hierarchical logistic regression analyses for the TR variables (mother and father reported CBCL-Int-TR, child reported MASC-TR and ADIS-TR). Higher levels of parent-reported assertion and self-control pre-treatment, but not responsibility, were significantly linked to a more favorable outcome on mother and father reported CBCL-TR-Int (see Tables 3 and 4). Likewise, higher levels of parent-reported assertion, responsibility and self-control pre-treatment were significantly linked to a more favorable outcome on ADIS-TR (see Table 5). None of the logistic regression analyses were significant for the MASC-TR (assertion; R^2^ < 0.01, χ^2^ = 0.02, *p* = 0.885, self-control; R^2^ = 0.02, χ^2^ = 1.42, *p* = 0.233; responsibility; R^2^ < 0.01, χ^2^ = 0.03, *p* = 0.863). This means that fathers and mothers reported a better treatment recovery on the CBCL in their children post-treatment if the child had higher levels of pre-treatment assertion and self-control. Also, the clinician reported a better treatment recovery on the ADIS if children had higher scores on all three social skills subscales (i.e., assertion, self-control, responsibility) pre-treatment. No such effects were found for children’s self-reported fear.

**Table 3:** Predictive value of assertion, self-control and responsibility for mother reported CBCL-TR-Int

Predictors	B	Exp(B)	R^2^	Model Chi^2^	*p*
Assertion					
Step 1			0.09	5.99	0.014
Constant	-4.41	< 0.01			0.000
Assertion	0.18	1.07			0.020
Step 2			0.14	3.69	0.158
Constant	-3.39	.03			0.018
Assertion	0.08	1.08			0.456
SoAP	2.75	15.66			0.056
Assertion x SoAP	-0.20	0.82			0.062
Self-control					
Step 1			0.14	10.07	0.002
Constant	-4.98	< 0.01			< 0.001
Self-control	0.29	1.34			0.004
Step 2			0.16	1.07	0.585
Constant	-4.23	.02			0.002
Self-control	0.21	1.24			0.080
SoAP	1.18	3.26			0.394
Self-control x SoAP	-0.12	0.89			0.338
Responsibility					
Step 1			0.06	3.71	0.054
Constant	-3.97	0.02			0.002
Responsibility	0.18	1.20			0.061
Step 2			0.08	1.37	0.505
Constant	-3.00	0.05			0.049
Responsibility	0.08	1.08			0.554
SoAD	1.76	5.82			0.250
Responsibility x SoAD	-0.16	0.86			0.244

*TR* Treatment-Recovery, *CBCL-TR-Int* Internalizing scale of the Child Behavior Checklist Treatment Recovery score

**Table 4:** Predictive value of assertion, self-control and responsibility for father reported CBCL-TR-Int

Predictors	B	Exp(B)	R^2^	Model Chi^2^	*p*
Assertion					
Step 1			0.16	5.56	0.018
Constant	-7.31	0.01			0.002
Assertion	0.30	1.36			0.031
Step 2			0.16	0.06	0.971
Constant	-6.91	< .01			0.030
Assertion	0.27	1.31			0.231
SoAP	0.79	2.20			0.805
Assertion x SoAP	-0.5	0.95			0.817
Self-control					
Step 1			0.16	5.82	0.016
Constant	-7.67	< 0.01			0.005
Self-control	0.42	1.53			0.044
Step 2			0.18	0.06	0.740
Constant	-6.79	< .01			0.024
Self-control	0.33	1.40			0.178
SoAP	0.97	2.63			0.747
Self-control x SoAP	-0.11	0.90			0.659
Responsibility					
Step 1			0.05	1.88	0.170
Constant	-5.44	< 0.01			0.016
Responsibility	0.22	1.25			0.183
Step 2			0.09	1.12	0.570
Constant	-3.45	0.03			0.226
Responsibility	0.01	1.01			0.966
SoAD	2.39	10.90			0.402
Responsibility x SoAD	-0.25	0.78			0.365

*TR* Treatment-Recovery, *CBCL-TR-Int* Internalizing scale of the Child Behavior Checklist Treatment Recovery score

**Table 5:** Predictive value of assertion, responsibility and self-control for ADIS-C/P Treatment Recovery and interaction with SoAD

Predictors	B	Exp(B)	R^2^	Model Chi^2^	*p*
Assertion					
Step 1			0.12	11.37	0.001
Constant	-0.18	15.24			0.001
Assertion	2.73	0.84			0.001
Step 2			0.16	3.99	0.136
Constant	2.35	10.51			0.013
Assertion	-0.14	0.87			0.047
SoAD	-1.88	0.15			0.049
Assertion x SoAD	.13	1.14			0.064
Self-control					
Step 1			0.11	10.04	0.002
Constant	2.20	9.00			0.003
Self-control	-0.19	0.83			0.002
Step 2			0.11	0.76	0.684
Constant	2.57	13.01			0.003
Self-control	-0.23	0.80			0.005
SoAD	0.72	2.05			0.412
Self-control x SoAD	-0.06	0.94			0.455
Responsibility					
Step 1			0.05	4.83	0.028
Constant	1.94	6.97			0.023
Responsibility	-0.14	0.84			0.032
Step 2			0.10	4.54	0.103
Constant	1.13	3.08			0.271
Responsibility	-0.06	0.95			0.532
SoAD	-2.13	0.12			0.037
Responsibility x SoAD	0.19	1.20			0.037

*TR* Treatment-Recovery, *SoAD* Social Anxiety Disorder

Next, we added SoAD and ‘social skills x SoAD’ as interaction term as a second step in the hierarchical regression analyses. For both father and mother CBCL-TR-Int scores, the significant main effects of assertion and self-control disappeared, and the interaction effect was not significant. Also, there were no significant (interaction) effects of the subscale responsibility (see Tables 3 and 4). For the MASC-TR, none of the regression analyses were significant (assertion; R^2^ = 0.01, χ^2^ = 1.13, *p* = 0.570, self-control; R^2^ = 0.02, χ^2^ = 0.25, *p* = 0.881, responsibility; R^2^ = 0.01, χ^2^ = 0.90, *p* = 0.637). For the ADIS-TR, the three main effects of social skills (assertion, self-control and responsibility) remained significant. In addition, we found a main effect of SoAD (*p* = 0.037) on responsibility, but this effect was qualified by a significant interaction effect of Responsibility x SoAD (*p* = 0.035; see Table 5). Children with no SoAD who had higher levels of responsibility pre-treatment had a more favorable outcome as measured with the ADIS-TR than children with lower social skills, but this effect did not show for children with SoAD (see Table 6).

**Table 6:** Means (*SD*) for responsibility for ADIS-C/P TR with SoAD

ADIS-C/P TR		
	Recovered	Not recovered
No SoAD	14.02 (2.63)	12.33 (2.67)^*^
SoAD	10.22 (2.43)	10.82 (2.04)

*TR* Treatment-Recovery, *SoAD* Social Anxiety Disorder

^*^p <0 .05 between recovered and not recovered

## Discussion

The current study investigated the role of social skills and its interaction effect with social anxiety disorder as predictors of treatment outcome. More specifically, the study had three goals 1) to examine whether, at pre-treatment, parents of children with SoAD reported significantly lower social skills in their children than parents of children without SoAD, 2) to explore the predictive value of social skills for treatment outcome and 3) to explore potential interaction effects of social skills and SoAD for treatment outcome. We assessed outcome in two different ways: Reliable Change (RC: The amount of reliable pre- to post-treatment change as measured with the MASC, and mother and father CBCL Internalizing problems), and Treatment-Recovery (TR: Treatment success versus treatment failure as measured by: Absence or presence of any anxiety disorder (ADIS-C/P); with mother and father reported Internalizing problems (CBCL) and with child reported anxiety (MASC)).

We found that parents of children with SoAD reported significantly lower levels of assertive and responsible social behavior prior to treatment in their children compared to parents of children with an anxiety disorder without SoAD. There are indeed several studies that found a clear link between the less favorable use of social skills and SoAD (Alfano et al., 2006; Dodd et al., 2011; Ginsburg et al., 1998; Inderbitzen-Nolan et al., 2007; Miers et al., 2010; Spence et al., 1999).

When looking at the second research question, that addressed the predictive value of social skills for treatment outcome, we did not find a significant effect of social skills on reliable change. Thus, children with an anxiety disorder can benefit from a generic treatment program regardless of the presence of parent-reported social skills difficulties. The results of the present study showed that higher levels of assertion, self-control and responsibility predicted a higher likelihood of Treatment-Recovery. This means that children with a higher level of pretreatment socially adequate behavior were more likely to be free of any anxiety disorder post treatment. When addressing the third goal of the study, by looking at the significant interaction of social skills with SoAD, we found that parental-reported responsibility was only a valid predictor for Treatment-Recovery in children without a SoAD, but not for children with SoAD. Children with no SoAD who had higher levels of responsibility had a more favorable outcome as measured with the ADIS-TR than children with lower social skills, but this effect did not show for children with SoAD. This effect was only found for the subscale responsibility, and not with the social skills subscales self-control and assertion. Moreover, it should be noted that even though the interaction between responsibility and social anxiety was significant, the overall interaction model was not significant, indicating that these results should be interpreted with care. In sum, it appears that higher social skills in general predicted Treatment-Recovery, and that SoAD only played a minor role in this relation. Notably, when looking at the interaction of parent-reported social skills and social anxiety predicting reliable change, the results showed that the children with anxiety, with and without social anxiety, profited equally regardless of their pretreatment level of parent-reported social skills.

These results could be explained by the fact that poor social skills might not necessarily be specific for social anxiety only (see also, Hofmann, 2000; Rapee & Heimberg, 1997). Poor social skills rather appear to pose a general vulnerability factor in the development of psychosocial problems (Segrin & Flora, 2000). Social skills did not predict the *amount* of pre- to post-treatment Reliable Change: Children with lower social skills benefited as much from the treatment as children with higher social skills, but these children were more impaired and needed relatively greater change compared to children with higher levels of social skills. Therefore, one could argue that we need to improve our generic cognitive behavioral treatment programs, by adding social skills training, especially for children with lower social skills. It should be noted however, that a social skills training might not result in socially important outcomes even when children acquire components of the necessary skills, if children are not able to integrate the newly learned skills into their daily behavioral repertoire (Greco & Morris, 2001). Likewise, the causal mechanism that accounts for parent-reported performance deficits of children with social anxiety in the current study could first be the result of social skills deficits (e.g., Dodd et al., 2011) or secondly, of difficulties using social skills due to anxiety (e.g., Spence & Rapee, 2016). Thirdly, social information processing theory typically accounts for how problems with social information processing results in socially inadequate behavior in children with externalizing behavior (De Castro, 2004). Notably, relations have also been found for social information processing with childhood anxiety (Luebbe et al., 2010). Consequentially, this has implications for treatment; either focus on addressing skills deficits in treatment with social skills training, decrease anxiety and thus enable the performance of skills, or address information processing deficits with (e.g.) cognitive restructuring.

When looking at the results in more detail, we found that parent-reported assertion, self-control and responsibility were significantly associated with treatment recovery (TR) but not with reliable change (RC). These findings underline the important role of the ability to be assertive, to respond in a responsible and self-controlled manner in social situations. Children with a behavioral ability to influence *social* events and *social* outcome’s in their environment (self-control), children who ask others for information, introduce themselves, and respond to actions of others (assertion) and children who communicate well with adults (responsibility) have a higher treatment recovery than children with scores on these subscales. Additionally, when looking at the interaction effects, these higher parent-reported social skills seemed to be partly only predictive for treatment recovery when children did not have SoAD. Thus, children with higher responsibility and no SoAD are most likely to recover from treatment (i.e., treatment success), but equally likely to profit from treatment (i.e., change during treatment). This might indicate that children with lower social skills might perhaps need 1) a more intensive treatment, for example by including more sessions, or may benefit from 2) the inclusion of social skills exercises. Clearly, more research is needed to shed more light on the role of social skills and social anxiety in treatment recovery, and to further study the possibilities to tailoring treatment programs. Knowledge on the underlying mechanisms or deviancies leading to lower social skills may hold keys to a more effective treatment for the subgroup of children with an anxiety disorder with lower social skills.

The current study had a few limitations. First, we only included parent-reported social skills of their children, but not therapist- or child-reported social skills. We also did not include any objective measures of social skills, for instance by asking the children to interact with their peers or to give a public speech task. Previous research has shown that self-reported levels of social skills may be negatively biased due to own levels of social anxiety, and that objective measures might give a better indication of social functioning in children with anxiety (Baartmans et al., 2019, 2020; Cartwright-Hatton et al., 2003; Klein et al., 2018; van Niekerk et al., 2017). Therefore, future studies should both include subjective and objective measures of social skills. Second, even though we had a relatively large sample of clinically anxious children in this study, only part of the children had a social anxiety disorder (*n* = 39; total *N* = 142). Furthermore, the current study used one specific treatment program, FRIENDS which relatively relies more on social support than other cbt-programs. It is important that more studies with even larger samples and using other treatment programs are conducted in order to verify the results found in the current study. Third, the SSRS that we used in the current study was a Dutch version of the original scale. Even though there was only one minor change compared to the original scale (i.e., one item was added to the subscale assertion), this new version was not formally validated. Fourth, the rate of participating fathers was lower compared to mothers which might have resulted in a lower power and a higher chance to find uneven distributions. For example, father reported CBCL-TR internalizing problems had one cell with an expected count below the threshold of 2,54, indicating that there was an uneven distribution in treatment recovery as indicated by the father. Therefore, these results must to be interpreted with caution. Finally, interrater reliability of the ADIS diagnoses was not calculated. Even though licensed therapists and Master students who were both supervised and trained in administering the interview, we have not systematically performed inter-rater reliabilities. As the current paper focuses on children with versus without social anxiety disorder in their diagnostic profile, it would have been important to include inter-rater reliabilities of these decisions.

In summary, the present study included a sample of children with an anxiety disorder with a relatively high rate of father and mother participation and a substantial proportion of children with SoAD. Whereas previous studies on the prediction of treatment outcome of cognitive behavioral therapy for children with anxiety disorders tended to overlook the social skills of children, the current study not only investigated the predictive value of social skills for treatment outcome, but also assessed interaction effects with social anxiety. Additionally, the current study also focused on statistically significant and meaningful change by examining treatment outcome with both reliable change and treatment recovery. The findings in the present study indicate that higher parent reported social skills in their children predicted higher treatment-recovery. Furthermore, there is some indication that children with higher levels of responsible behavior show a better treatment recovery, but not for children with a social anxiety disorder. These results underline the relevance of further investigation the role of social skills for treatment outcome of children with anxiety disorders.

## Key Points of the Paper

- Parents of anxious children with social anxiety disorder (SoAD) report lower levels of assertion and responsibility than parents of anxious children without SoAD
- Higher level of social skills predicts a higher likelihood of treatment-recovery in children with anxiety treated with a CBT program
- Higher level of social skills does not predict the amount of pre- to post-treatment reliable change
- The association between social skills and treatment-recovery is only partly moderated by SoAD
- Children with an anxiety disorder with and without SoAD may benefit equally from a generic CBT program

## References

[CR1] Achenbach TM (1991). Integrative guide for the CBCL/4-18, YSR, and TRF profiles.

[CR2] Alfano CA, Beidel DC, Turner SM (2006). Cognitive correlates of social phobia among children and adolescents. Journal of Abnormal Child Psychology.

[CR3] Baartmans JM, Rinck M, Hudson JL, Lansu TA, van Niekerk RE, Bögels SM, Klein AM (2019). Are socially anxious children really less liked, or do they only think so?. Cognitive Therapy and Research.

[CR4] Baartmans, J. M., van Steensel, F. J., Mobach, L., Lansu, T. A., Bijsterbosch, G., Verpaalen, I., & Klein, A. M. (2020). Social anxiety and perceptions of likeability by peers in children. *British Journal of Developmental Psychology, **38*, 319–336. 10.1111/bjdp.12324 PMC7216937 32064647

[CR5] Barrett PM, Turner C (2000). Friends for children: Group leader's manual.

[CR6] Cartwright-Hatton S, Hodges L, Porter J (2003). Social anxiety in childhood: The relationship with self and observer rated social skills. Journal of Child Psychology and Psychiatry.

[CR7] Cartwright-Hatton S, Roberts C, Chitsabesan P, Fothergrill C, Harrington R (2004). Systematic review of the efficacy of cognitive behaviour therapies for childhood and adolescent anxiety disorders. British Journal of Psychiatry.

[CR8] Cartwright-Hatton S, Tschernitz N, Gomersall H (2005). Social anxiety in children: Social skills deficit, or cognitive distortion?. Behaviour Research and Therapy.

[CR9] Chambless DL, Hollon SD (1998). Defining empirically supported therapies. Journal of Consulting and Clinical Psychology.

[CR10] Creswell, C., Nauta, M. H., Hudson, J. L., March, S., Reardon, T., Arendt, K., & In‐Albon, T. (2020). Research Review: Recommendations for reporting on treatment trials for child and adolescent anxiety disorders–an international consensus statement. *Journal of Child Psychology and Psychiatry*. 10.1111/jcpp.13283 32683742

[CR11] De Castro BO (2004). The development of social information processing and aggressive behaviour: Current issues. European Journal of Developmental Psychology.

[CR12] de Groot A, Koot HM, Verhulst FC (1994). Cross-Cultural Generalizability of the Child Behavior Checklist Cross-Informant Syndromes. Psychological Assessment.

[CR13] De Winter AF, Oldehinkel AJ, Veenstra R, Brunnekreef JA, Verhulst FC, Ormel J (2005). Evaluation of non-response bias in mental health determinants and outcomes in a large sample of pre-adolescents. European Journal of Epidemiology.

[CR14] Dodd H, Hudson J, Lyneham H, Wuthrich V, Morris T, Monier L (2011). Biased self-perception of social skills in anxious children: The role of state anxiety. Journal of Experimental Psychopathology.

[CR15] Emmelkamp, P. M. G., Ehring, T., & Powers, M.B. (2010). Philosophy, psychology, causes and treatments of mental disorders. In N. Kazantzis, M.A. Reinecke, & A. Freeman (Eds.), *Cognitive and behavioral theories in clinical practice* (pp. 1–27). New York: Guilford.

[CR16] Fréchette-Simard C, Plante I, Bluteau J (2018). Strategies included in cognitive behavioral therapy programs to treat internalized disorders: A systematic review. Cognitive Behaviour Therapy.

[CR17] Ginsburg GS, La Greca AM, Silverman WK (1998). Social anxiety in children with anxiety disorders: Relation with social and emotional functioning. Journal of Abnormal Child Psychology.

[CR18] Greco LA, Morris TL (2001). Treating childhood shyness and related behavior: empirically evaluated approaches to promote positive social interactions. Clinical Child and Family Psychology Review.

[CR19] Gresham FM, Elliott SN (1990). Social skills rating system: Manual.

[CR20] Gresham FM (2016). Social skills assessment and intervention for children and youth. Cambridge Journal of Education.

[CR21] Hageman WJ, Arrindell WA (1999). Clinically significant and practical! Enhancing precision does make a difference. Reply to McGlinchey and Jacobson, Hsu, and Speer. Behavior Research and Therapy.

[CR22] Hebert-Myers H, Guttentag CL, Swank PR, Smith KE, Landry SH (2006). The importance of language, social, and behavioral skills across early and later childhood as predictors of social competence with peers. Applied Developmental Science.

[CR23] Hofmann SG (2000). Treatment of social phobia: Potential mediators and moderators. Clinical Psychology.

[CR24] Hopko DR, McNeil DW, Zvolensky MJ, Eifert GH (2001). The relation between anxiety and skill in performance-based anxiety disorders: A behavioral formulation of social phobia. Behavior Therapy.

[CR25] Hudson JL, Keers R, Roberts S, Coleman JR, Breen G, Arendt K, Eley TC (2015). Clinical predictors of response to cognitive-behavioral therapy in pediatric anxiety disorders: The genes for treatment (GxT) study. Journal of the American Academy of Child and Adolescent Psychiatry.

[CR26] Hudson JL, Rapee RM, Lyneham HJ, MacLellan LF, Wuthrich VM, Schniering CA (2015). Comparing outcomes for children with different anxiety disorders following cognitive behavioural therapy. Behaviour Research and Therapy.

[CR27] Inderbitzen-Nolan HM, Anderson ER, Johnson HS (2007). Subjective versus objective behavioral ratings following two analogue tasks: A comparison of socially phobic and non-anxious adolescents. Journal of Anxiety Disorders.

[CR28] Jacobson NS, Roberts LJ, Berns SB, McGlinchey JB (1999). Methods for defining and determining the clinical significance of treatment effects: Description, application, and alternatives. Journal of Consulting and Clinical Psychology.

[CR29] Jacobson NS, Truax P (1991). Clinical significance: A statistical approach to defining meaningful change in psychotherapy research. Journal of Consulting and Clinical Psychology.

[CR30] James, A. C., James, G., Cowdrey, F. A., Soler, A., & Choke, A. (2013). Cognitive behavioural therapy for anxiety disorders in children and adolescents. *Cochrane **Database of Systematic Reviews, 6*. 10.1002/14651858.CD004690.pub3 23733328

[CR31] Kazdin AE (1999). The meanings and measurement of clinical significance. Journal of Consulting and Clinical Psychology.

[CR32] Khanna MS, Kendall PC (2010). Computer-assisted cognitive-behavioral therapy for child anxiety: Results of a randomized clinical trial. Journal of Consulting and Clinical Psychology.

[CR33] Klein AM, Houtkamp EO, Salemink E, Baartmans JMD, Rinck M, van der Molen MJ (2018). Differences between self- and peer-rated likability in relation to social anxiety and depression in adolescents with mild intellectual disabilities. Research in Developmental Disabilities.

[CR34] Kodal A, Fjermestad K, Bjelland I, Gjestad R, Öst L-G, Bjaastad JF, Haugland BSM, Havik OE, Heiervang E, Wergeland GJ (2018). Long-term effectiveness of cognitive behavioral therapy for youth with anxiety disorders. Journal of Anxiety Disorders.

[CR35] Liber JM, van Widenfelt BM, Utens EMWJ, Ferdinand RF, van der Leeden AJM, van Gastel W, Treffers PDA (2008). No differences between group versus individual treatment of childhood anxiety disorders in a randomized clinical trial. Journal of Child Psychology and Psychiatry.

[CR36] Luebbe AM, Bell DJ, Allwood MA, Swenson LP, Early MC (2010). Social information processing in children: Specific relations to anxiety, depression, and affect. Journal of Clinical Child & Adolescent Psychology.

[CR37] Lundkvist-Houndoumadi I, Thastum M (2017). Anxious children and adolescents non-responding to CBT: Clinical predictors and families’ experiences of therapy. Clinical Psychology & Psychotherapy.

[CR38] March JS (1997). Multidimensional anxiety scale for children: Technical manual.

[CR39] March JS, Sullivan K, Parker JDA (1999). Test-retest reliability of the multidimensional anxiety scale for children. Journal of Anxiety Disorders.

[CR40] McDaniel, S. C., Bruhn, A. L., & Troughton, L. (2017). A brief social skills intervention to reduce challenging classroom behavior. *Journal of Behavioral Education 26*, 53–74.

[CR41] McLeod, B. M., Jensen-Doss, A., & Ollendick, T. H. (2013). Overview of diagnostic and behavioral assessment. In McLeod, B.M., Jensen-Doss, A., & Ollendick, T. H. (Eds.), *Diagnostic and Behavioral Assessment in Children and Adolescents. A Clinical Guide (pp. 1–33).* The Guilford Press, New York.

[CR42] Miers AC, Blöte AW, Westenberg PM (2010). Peer perceptions of social skills in socially anxious and nonanxious adolescents. Journal of Abnormal Child Psychology.

[CR43] Nauta MH, Scholing A, Emmelkamp PM, Minderaa RB (2003). Cognitive behavioral therapy for children with anxiety disorders in a clinical setting: No additional effect of a cognitive parent training. Journal of the American Academy of Child & Adolescent Psychiatry.

[CR44] Rapee RM (2000). Group treatment of children with anxiety disorders: Outcome and predictors of treatment response. Australian Journal of Psychology.

[CR45] Rapee RM, Heimberg RG (1997). A cognitive-behavioral model of anxiety in social phobia. Behaviour Research and Therapy.

[CR46] Rapee RM, Schniering CA, Hudon JL (2009). Anxiety disorders during childhood and adolescence: Origins and treatment. Annual Review of Clinical Psychology.

[CR47] Segrin C, Flora J (2000). Poor social skills are a vulnerability factor in the development of psychosocial problems. Human Communication Research.

[CR48] Shortt AL, Barrett PM, Fox TL (2001). Evaluating the FRIENDS Program: A cognitive-behavioral group treatment for anxious children and their parents. Journal of Clinical Child Psychology.

[CR49] Siebelink BM, Treffers PDA (2001). *ADIS-C Anxiety Disorders Interview Schedule for DSM-IV* (Dutch Version).

[CR50] Silverman WK, Albano AM (1996). The Clinician Manual for the Anxiety Disorders Interview Schedule for DSM-IV.

[CR51] Silverman WK, Kurtines WM, Ginsburg GS, Weems CF, Rabian B, Serafini LT (1999). Contingency management, self-control, and education support in the treatment of childhood phobic disorders: A randomized clinical trial. Journal of Consulting and Clinical Psychology.

[CR52] Silverman WK, Saavedra LM, Pina AA (2001). Test-retest reliability of anxiety symptoms and diagnoses with the Anxiety Disorders Interview Schedule for DSM-IV: child and parent versions. Journal of the American Academy of Child & Adolescent Psychiatry.

[CR53] Spence SH, Donovan C, Brechman-Toussaint M (1999). Social skills, social outcomes and cognitive features of childhood social phobia. Journal of Abnormal Psychology.

[CR54] Spence, S. H., & Rapee, R. M. (2016). The etiology of social anxiety disorder: An evidence-based model. *Behaviour Research and Therapy, 86*, 50–67. 10.1016/j.brat.2016.06.007 27406470

[CR55] Utens, E. M. W. J., de Nijs, P., & Ferdinand, R. F. (2001). *FRIENDS for children- manual for group leaders (Dutch translation)*. Department of Child and Adolescent Psychiatry Erasmus MC- Sophia Childrens Hospital Rotterdam, Rotterdam, NL.

[CR56] Utens EMWJ, Ferdinand RF (2000). Multidimensional Anxiety Scale for Children (MASC Dutch translation: MASC-NL).

[CR57] van Niekerk RE, Klein AM, Hudson JL, Allart E, Rinck M, Becker ES (2017). The role of cognitive factors in social anxiety: Social threat thoughts and social skills perception in children. Cognitive Research and Therapy.

[CR58] Verhulst FC, van der Ende J, Koot HM (1996). Manual for the Child Behavior Checklist (in Dutch).

[CR59] Victor AM, Bernat DH, Bernstein GA, Layne AE (2006). Effects of parent and family characteristics on treatment outcome of anxious children. Journal of Anxiety Disorders.

[CR60] Waters AM, Groth TA, Purkis H, Alston-knox C (2018). Predicting outcomes for anxious children receiving group cognitive-behavioural therapy: Does the type of anxiety diagnosis make a difference?. Clinical Psychologist.

